# Current Evidence on Long-Term Prognostic Factors in Vasospastic Angina

**DOI:** 10.3390/jcm10184270

**Published:** 2021-09-21

**Authors:** Hack-Lyoung Kim, Sang-Ho Jo

**Affiliations:** 1 Division of Cardiology, Department of Internal Medicine, Boramae Medical Center, National University College of Medicine, Seoul 07061, Korea; khl2876@gmail.com; 2 Division of Cardiology, Department of Internal Medicine, Hallym University Sacred Heart Hospital, Anyang 14068, Korea

**Keywords:** calcium channel blocker, coronary spasm, prognosis, prognostic factors, vasospastic angina

## Abstract

Vasospastic angina (VSA) is characterized by a reversible spasm of the coronary arteries and is more prevalent in Asians. Vasodilators, such as calcium channel blockers, are effective in relieving coronary spasms and preventing clinical events. Therefore, the prognosis of VSA is generally known to be better than for significant organic stenosis caused by atherosclerosis. However, coronary vasospasm is sometimes associated with fatal complications such as sudden death, ventricular arrhythmia, and myocardial infarction. Thus, it is very important to identify and actively treat high-risk patients to prevent VSA complications. Here, we will review clinical factors associated with long-term prognosis in patients with VSA.

## 1. Introduction

In 1959, Dr. Myron Prinzmental was the first to describe a new form of exercise-independent angina, and named it a “variant form of angina pectoris”, which is now more commonly referred to as vasospastic angina (VSA) [1]. VSA is characterized by transient myocardial ischemia due to dynamic coronary artery spasm, resulting in the clinical presentation of chest pain and electrocardiographic changes [2,3,4]^)^. Coronary spasm is relatively well relieved by vasodilators such as calcium channel blockers (CCBs). These drugs effectively prevent the occurrence of clinical events associated with a coronary spasm, and thus, VSA usually has a favorable long-term prognosis compared to atherosclerotic coronary stenosis [4,5,6]. Therefore, vasodilators are the cornerstone of treatment for VSA [7]. However, despite the use of vasodilators, some patients with VSA develop serious cardiac complications, such as sudden cardiac death, myocardial infarction, and ventricular arrhythmias due to a severe coronary spasm [8,9,10]^)^. Considering that the ultimate goal of the treatment for VSA patients is to prevent the occurrence of such fatal complications, it is very important to find the factors that determine the prognosis of VSA patients. Knowing these prognostic factors will also lead to a better understanding and management of VSA. However, there are limited data on the prognostic factors of VSA. As the prevalence of VSA is low, and it mainly occurs in Asians, relatively few studies have been conducted, especially in Japan [11]. However, it has been suggested that VSA incidence was also higher in Westerners than ever considered [12]. In recent years, the awareness and interest in VSA have gradually increased in both the East and the West, and several reports that investigate the risk factors determining the long-term prognosis of VSA have been published. However, because each study has different patient characteristics, the risk factors associated with the long-term prognosis for VSA were various and sometimes conflicting. Comprehensive assessment and summary of the risk factors for long-term clinical outcomes of VSA, including current evidence, will be of great help to many clinicians managing VSA patients and researchers interested in VSA.

## 2. Prognosis of Patients with VSA

VSA prognosis is generally known to be not so bad if the patient is on medication and does not smoke or drink. Waters et al. investigated 169 consecutive Canadian VSA patients and demonstrated that their survival at 1, 2, and 3 years was 95%, 90%, and 87%, and survival without myocardial infarction was 80%, 78%, and 75%, respectively [13]. In a Japanese study with a long-term clinical follow-up of 245 VSA patients, the survival rate at 1, 3, 5, and 10 years was 98%, 97%, 97%, and 93%, respectively, and survival rate without myocardial infarction at 1, 3, 5, and 10 years was 86%, 85%, 83% and 81%, respectively [5]. Bory et al. examined 277 VSA patients in France with a median follow-up of 7.4 years and found that all-cause and cardiac mortality rates were 7.2% and 3.6%, respectively [14]. Shimokawa et al., who investigated 1244 VSA Japanese patients, revealed that the incidence of all-cause and cardiac mortality during 2.7 years of follow-up were 1.3% and 0.3%, respectively [15]. In Japan’s national registry data, the 5-year survival rate of VSA patients was 94% for men and 93% for women [16]. More recently, a Korean study including 2129 patients with coronary spasms demonstrated that the 2-year incidence of cardiac death was 0.9% [17]. Collectively, although VSA prognosis varies according to studies due to different patients’ characteristics and follow-up duration, the prognosis for VSA is generally better compared to typical angina caused by fixed organic stenosis of the epicardial coronary artery.

## 3. Factors Associated with Long-Term Prognosis of VSA

### 3.1. Age and Sex

Many studies have shown that older age is associated with poor clinical outcomes in patients with VSA [18,19,20]. Older patients usually have more comorbidities and organic stenosis [21]. Kim et al. indicated that, among various clinical factors, only old age was an independent factor associated with rehospitalization for VSA in multivariable analysis [20]. It has been reported that young age (<50 years) was associated with better clinical outcomes in female VSA patients but not in male VSA patients, which suggests the important role of female sex hormones in cardiovascular protective effects [16]. However, unlike these studies, in some studies, age was not related to the long-term prognosis in patients with VSA [22,23]. Another study, rather, has reported that older age is associated with a better prognosis of VSA [24]. Age may be less influential as a prognostic factor in patients with VSA compared to patients with significant organic stenosis [25].

The VSA prognosis is expected to be worse in men than in women because men smoke and drink more and have a higher prevalence of accompanying organic stenosis [16,22,26]. However, results from prior studies on this issue were different from our expectations. Several recent studies have focused on sex differences, in which the long-term cardiovascular prognosis was similar between sexes in patients with VSA [16,22,26]. Although not focused on sex differences, other studies that analyzed patients with VSA have also consistently shown that sex is not associated with the long-term prognosis [5,18,23,27,28,29]. Pathophysiology on why the baseline clinical characteristics unfavorable to men are not reflected in the long-term prognosis of VSA is unknown and requires further studies.

### 3.2. Obesity

In the nation-wide prospective registry of patients with VSA in Korea (VA-KOREA registry), overweight or obesity was associated with a lower occurrence of clinical events during a 1-year follow-up of VSA patients compared to patients with normal body weight, and the authors suggested the existence of an “obesity paradox” in VSA [30]. Kim et al. showed that obesity (body mass index ≥ 25 kg/m^2^) was associated with a better prognosis in female VSA patients; however, this association was not observed in male VSA patients [31]. There are still insufficient data on the association between body mass index and the long-term prognosis of patients with VSA.

### 3.3. Smoking and Alcohol

Smoking provokes coronary artery spasms [31,32]. Smoking is a well-known stressor to vasculature by causing blood pressure elevation, abnormal lipid metabolism, insulin resistance, oxidative stress, and endothelial cell dysfunction [33]. Choi et al. assessed long-term clinical events in 1590 VSA patients according to their smoking status and showed that major adverse cardiovascular event (MACE) incidence was not associated with smoking. However, the same authors demonstrated that smokers with VSA experienced a significantly higher incidence of recurrent angina compared to non-smokers with VSA during the 3-year follow-up period [34]. Although not focused on smoking, several other studies have shown that smoking is an independent factor in determining long-term prognosis in patients with VSA [17,35,36,37]. In a 12-year follow-up study of 273 patients with VSA in Spain, smoking was associated with a 2.4-fold increased mortality risk [36]. However, smoking was not associated with cardiac mortality or myocardial infarction in the same study. In a Japanese study of 1429 VSA patients, smoking increased the incidence of MACE by 1.4 times at a 32-month clinical follow-up [35]. Cho et al. demonstrated that smoking was associated with higher rates of unfavorable cardiovascular outcomes, especially in patients taking antiplatelet agents [38]. On the other hand, other studies have shown that smoking was not associated with the long-term prognosis of patients with VSA [5,22,26].

Alcohol is another well-known risk factor for coronary artery spasms [4,39,40,41]. Alcohol is known to induce an inflammatory response, increase free radical production, and decrease nitric oxide production, leading to coronary spasm [39,41]. However, little is known about the effects of alcohol on the prognosis of patients with VSA. In a recent Korean study, although excessive alcohol consumption was a stronger risk factor for coronary artery spasm, it was not associated with long-term clinical outcomes in patients with VSA [41]. Other studies also have shown a lack of association between alcohol consumption and long-term clinical outcome of VSA [29]. Additional clinical evidence is needed to more clearly understand the association between alcohol and the long-term prognosis of VSA patients.

The evidence for the role of smoking and alcohol as long-term prognostic factors in patients with VSA is not strong. This is probably because intensive medical therapy with vasodilators effectively reduces VSA attacks caused by smoking or alcohol drinking. However, both smoking and alcohol clearly cause coronary spasms, so it should always be emphasized to maintain strict drug intake along with smoking cessation and alcohol abstinence in patients with VSA.

### 3.4. Traditional Risk Factors

Traditional cardiovascular risk factors, such as hypertension, diabetes mellitus, or dyslipidemia, are major factors that exacerbate coronary atherosclerosis and develop cardiovascular events [42,43]. However, these traditional risk factors do not appear to significantly affect the long-term prognosis of patients with VSA. Some studies have shown a significant association between traditional risk factors and the long-term prognosis of VSA [14,18,29]. In a European study in which 277 VSA patients were followed for 89 months, the risk factors for major coronary events in these patients were hypertension and the presence of coronary luminal irregularity [14]. In the study of Kim et al., only hypertension was associated with the long-term prognosis of patients with VSA among hypertension, diabetes, and dyslipidemia [18]. Han et al. performed 36 months of clinical follow-up of 1786 VSA patients and demonstrated that hypertension and dyslipidemia were associated with 3.3- and 5.1-fold higher MACE rates, respectively [29]. However, a larger number of other studies have reported that these traditional cardiovascular risk factors were not associated with VSA prognosis [22,23,35,37,44]. As there are no studies focused on traditional risk factors, and the results are different among studies, additional studies are needed to confirm the role of traditional risk factors on the prognosis of patients with VSA.

### 3.5. Clinical Presentation

The clinical manifestations of coronary artery spasms are diverse, including angina pectoris, myocardial infarction, life-threatening arrhythmia, and cardiac arrest [3,4]. ST-segment elevation during an attack was a significant predictor of survival [5,35,45]. Acute coronary syndrome (ACS) presentation in VSA patients was associated with an increased risk of MACE and recurrent myocardial infarction in comparison to those without ACS [46].

The long-term prognosis of patients with cardiac arrest caused by VSA was poorer than that of patients with VSA who did not develop cardiac arrest [9,35]. Takagi et al. showed that a survival rate free from MACE was significantly lower in VSA patients with cardiac arrest than those without (72% vs. 92% at 5 years). Multivariate analysis in the same study showed that the risk of developing MACE in patients with cardiac arrest was 3.26 times that of patients without cardiac arrest [35]. In the same study, there were two implantable cardioverter defibrillator (ICD) shock deaths and one cardiac death among 35 VSA patients with aborted sudden cardiac death (ASCD) during a median 2.7-year follow-up. More recently, an observational study of 188 VSA patients with ASCD and 1844 VSA patients without ASCD revealed that the incidence of cardiac death was significantly higher in patients with ASCD than those without over a median follow-up of 7.5 years (hazard ratio (HR), 7.26; 95% CI, 4.21–12.5) [9]. These studies suggest that VSA patients who had a history of cardiac arrest, even if they completely recovered from the cardiac arrest, should be considered very-high-risk patients because they have high rates of future cardiovascular events and mortality. However, another observational study of 18 VSA patients with ASCD reported favorable outcomes in these patients by showing no fatal arrhythmia or cardiac arrest during a median follow-up of 2.8 years [47].

Since one of the important factors affecting VSA prognosis is the occurrence of ventricular fibrillation, it has been suggested that ICD should be implanted for secondary prevention [3,48]. Although several studies have reported that ICD therapy improved the prognosis of patients with VSA who survived cardiac arrest, these are retrospective or observational studies, and the number of enrolled patients is small [48,49,50]. Further well-designed studies with larger sample sizes are needed to determine whether ICD therapy improves the prognosis of patients with VSA and history of cardiac arrest. In Korea, RCT is currently underway to determine whether ICD therapy is effective in improving the prognosis of VSA patients manifesting as ASCD (ClinicalTrials.gov Identifier: NCT02845531). The RCT is expected to close in 2023, and its results could provide important guidance to clinicians.

### 3.6. Angiographic Findings

A substantial portion of VSA patients also have coronary artery stenosis caused by atherosclerosis [17]. Coronary spasm more frequently occurs in a segment with coronary atherosclerosis because of endothelial dysfunction and abnormal vasomotor function [27,51]. Therefore, combined organic stenosis is one of the important prognostic factors for patients with VSA. Many studies have shown that the extent and severity of combined atherosclerotic coronary artery disease (CAD) was one of the independent prognostic factors of cardiovascular outcome in VSA patients [5,13,17,22,24,37]. In a study that followed 169 VSA patients, survival for patients with multi-vessel disease was 81%, 76%, and 66%; for patients with one-vessel disease, 97%, 92%, and 92%; and for patients without significant stenosis, 98%, 98%, and 98% at 1, 2, and 3 years, respectively [13]. Kim et al. analyzed 1920 VSA patients and found that significant stenosis was an independent risk factor for long-term prognosis (1-year MACE rate: 5.8% vs. 1.4%) [24]. In the sex-specific analysis using the same database, combined organic stenosis was significantly associated with long-term clinical outcomes in both male and female patients with VSA [22]. According to the results of a recent study of 175 Japanese patients with VSA, those with organic stenosis with a fractional flow reserve of ≤0.80 showed a higher MACE rate compared to those without organic stenosis during a median follow-up period of 656 days (37.6% vs. 3.6%) [37].

Shin et al. analyzed 2129 VSA patients from the VA-KOREA (Vasospastic Angina in Korea) registry and demonstrated that 24-month incidences of cardiac death, arrhythmia, and ACS were significantly higher in patients with positive test results in the intracoronary ergonovine provocation test (>90% luminal narrowing) than those with intermediate results (50~90% luminal narrowing) [17]. In concordance with this finding, Sunagawa et al. showed that patients with an intermediate coronary spasm in the ergonovine provocation test had a similarly good prognosis compared to those with a negative provocation test [52]. Additionally, it has been reported that the occurrence of cardiovascular events was significantly higher in VSA patients with a multi-vessel spasm during the ergonovine provocation test than the single-vessel VSA and non-VSA group [28,29,35,53]. A current Japanese study demonstrated that an acetylcholine-induced diffuse coronary spasm was associated with a better prognosis than focal spasm [54]. Consistent with this finding, Kim et al. showed that patients with a focal spasm induced by ergonovine had a significantly higher 2-year incidence of cardiac events, including cardiac death, ACS, and arrhythmia, than in those with a diffuse spasm [55]. Similar findings were also found in other studies [56,57]. It has been reported that long-term prognosis was worse in VSA patients with a coronary spasm that occurred at the site of organic stenosis compared to those with a coronary spasm occurring at a site other than the site of a significant organic stenosis [5,22,58]. A coronary artery spasm is common in the segment of myocardial bridging because it is frequently accompanied by endothelial cell dysfunction [59,60,61]. A Korean study reported that combined myocardial bridging in patients with VSA was associated with a higher incidence of recurrent angina, but not with MACE compared to those with only myocardial bridging [61]. There are still very little research data on the effect of myocardial bridging on the long-term prognosis of VSA patients.

The results of these studies suggest that a provocation test with invasive coronary angiography is needed to identify the location and characteristics of coronary artery spasms. Also, the use of anti-atherosclerotic medications, such as antiplatelet agents and statins, should be actively considered for the treatment of not only coronary artery spasms but also associated organic stenosis.

### 3.7. Medications

In a study that followed up 245 VSA patients for an average of 80 months, the use of CCB was the most important factor in determining the long-term survival rate of patients [5]. Another study found that CCB did not affect the prognosis of patients with VSA with multi-vessel CAD but was a significant prognostic factor in patients without multi-vessel CAD [13]. A recent Korean study showed that the type of CCB (first generation (diltiazem and nifedipine) vs. second generation (amlodipine and benidipine) CCBs) did not affect the long-term prognosis of VSA patients [62]. In addition, adherence to medication is an important prognostic factor in VSA. If VSA patients do not take adequate medications such as CCB, the prognosis is poor because VSA attacks are more severe and frequent compared to those with good medication adherence [18,35,63].

Nitrates are widely used in patients with VSA as a drug to relieve coronary spasms, but the effect of the long-term use of this drug on the prognosis of patients with VSA is not well-known.

There has always been concern about the intolerance and potentially harmful effects of the long-term use of nitrates on the cardiovascular system [64]. In this regard, several studies have reported that the long-term use of nitrates was associated with a higher incidence of cardiovascular events in patients with VSA [58,64,65,66]. However, most of these studies are observational or retrospective rather than randomized studies. Therefore, well-designed studies are needed to clarify the prognosis of patients with VSA after long-term use of nitrates.

Stain is widely used for CAD caused by atherosclerosis due to its anti-inflammatory, anti-oxidant, endothelial-protective, and plaque-stabilizing actions, as well as cholesterol-lowering effect [67,68]. Considering that coronary artery spasms often occur in lesions accompanied by atherosclerosis, it is inferred that statins with such strong anti-atherosclerotic actions will have a clear beneficial effect even in VSA. Yasue et al. showed that adding a statin to CCB significantly reduced the incidence of acetylcholine-induced coronary spasm as compared with CCB-only therapy [69]. In a study of 1402 Japanese patients with VSA, statin use was associated with a better 5-year, MACE-free survival rate [45]. However, in a more recent study of 1713 Korean patients with VSA, the use of statins was not related to the long-term prognosis of these patients [57]. Another Korean study also demonstrated that statin therapy did not improve long-term clinical outcomes in VSA patients without significant organic stenosis [70]. Consistent with these findings, in a study analyzing 4099 VSA patients admitted to the intensive care unit from the national health insurance data, statin use was not associated with myocardial infarction or cardiac arrest events during clinical follow-up for 3.8 years [71]. Considering the results of these studies, it is still questionable whether statins have an additive effect on the long-term prognosis of VSA patients in addition to the effects of CCB and nitrates.

Several studies have shown that aspirin could aggravate coronary artery spasms [72,73]. It has been suggested that decreased production of endothelial prostaglandin in the coronary artery may be associated with coronary artery spasms [72]. Some studies have shown the impact of aspirin on the long-term clinical outcome of VSA. A recent study performed by Lim et al. investigated 777 Korean patients with VSA without significant coronary artery stenosis and reported that patients taking low-dose aspirin were at higher risk of MACE, mainly due to increased rehospitalization [74]. Another Korean study reported that the use of dual antiplatelet therapy with aspirin and clopidogrel in patients with VSA and ACS was associated with a poorer long-term clinical outcome than in those with aspirin alone or in those who received no antiplatelet agents [75]. In that study, aspirin use alone was not associated with a worse clinical outcome. A Japanese study performed by Ishii et al. also demonstrated that there was no difference in MACE incidence between patients with and without low-dose aspirin among VSA patients without significant organic stenosis [76]. Consistent with these findings, a recent meta-analysis of 3661 VSA patients in four studies found that aspirin use in patients without organic stenosis was not associated with myocardial infarction or cardiac death at clinical follow-up for 1 to 5 years [77].

In summary, aspirin and statin use is not necessary to improve the long-term prognosis of patients with VSA unless significant stenosis is combined.

### 3.8. C-Reactive Protein

C-reactive protein (CRP) is a marker of chronic inflammation and is associated with the prognosis of various cardiovascular diseases. Several studies have reported that CRP is elevated in patients with VSA and that CRP concentration is useful in VSA diagnosis [78,79,80]. Cho et al. showed that elevated CRP level was associated with the clinical presentation of ACS in patients with VSA [46]. However, there are few studies on how CRP affects the long-term prognosis of patients with VSA, and the results are still conflicting. Hung et al. followed up 897 patients who underwent an ergonovine provocation test, and found that high CRP levels were associated with more coronary events during 4 years of clinical follow-up [23]. Care should be taken when interpreting the results of this study because only 57% of patients had confirmed VSA, and other confounding variables affecting prognosis were not corrected. Another study suggested sex-specific values of CRP by showing that baseline CRP levels were an independent predictor of cardiovascular events in male patients with VSA, but not in female patients with VSA [26]. Different from these results, Park et al. found that although CRP had a diagnostic value for VSA, baseline CRP levels were not associated with a 1-year clinical outcome in the patients who underwent acetylcholine provocation tests [78]. Further studies are needed on the prognostic value of CRP in VSA patients.

### 3.9. Others

Low SES is associated with poor cardiovascular outcomes in patients with atherosclerotic cardiovascular disease [81,82,83]. It has been suggested that individuals with low SES have less access to health care and have a harder time maintaining a healthy lifestyle compared to those with high SES [19]. However, data on whether SES can affect the prognosis of patients with VSA have been scarcely reported. Recently, Kim et al. investigated 2476 VSA patients from the National Health Insurance Service—National Sample Cohort and demonstrated that low household income was significantly associated with all-cause (adjusted HR = 1.52 for highest vs. lowest SES) and cardiac (adjusted HR = 1.80 for highest vs. lowest SES) mortality during a 5-year clinical follow-up even after controlling the effects of potential confounders [18]. This study highlights the importance of the continuity of monitoring and treatment for those with low SES among VSA patients.

A Japanese researcher recently presented a scoring system for predicting the long-term prognosis of 1,429 Japanese patients with VSA [28]. In that study, the authors suggested the following seven risk factors for VSA prognosis: history of out-of-hospital cardiac arrest, smoking, angina at rest alone, organic stenosis, multi-vessel spasm, ST-segment elevation during angina, and the use of beta-blocker. Patients with more of these risk factors had a poorer prognosis for VSA: MACE (cardiac death, nonfatal myocardial infarction, unstable angina, heart failure, and appropriate ICD shock) rates were 2.5%, 7.0%, and 13.0% in patients with a low risk score (0–2), intermediate risk score (3–5), and high risk score (≥6), respectively [28].

## 4. Conclusions

Factors associated with long-term prognosis of patients with VSA were summarized in Table 1. Compared to CAD caused by atherosclerosis, data on the long-term prognosis of VSA is limited. However, several studies have consistently shown that more serious clinical presentations such as ST elevation, ACS and cardiac arrest, combined organic stenosis, definite spasms in provocation tests, focal spasms, multi-vessel spasms, and not using CCB are independent risk factors associated with long-term clinical outcomes of patients with VSA. Many of these variables that determine a patient’s long-term prognosis can be obtained through invasive coronary angiography and provocation tests. Therefore, in order to select high-risk patients and provide customized treatment, invasive angiography and provocation tests should be actively performed when VSA is suspected. In addition, continuing anti-atherosclerotic treatments for accompanying organic stenosis and maintaining CCB intake are important for improving the long-term prognosis of patients with VSA. Although the effects of smoking and alcohol drinking on the long-term prognosis of patients with VSA are controversial, it is clear that they cause coronary spasms and should be avoided. Nitrates effectively relieve coronary spasms in acute settings; however, it should be kept in mind that the long-term use of long-acting nitrates can adversely affect the prognosis of VSA patients. Several clinical variables that are suggested as long-term prognostic factors in some studies require further investigation to confirm their roles in VSA.

## Figures and Tables

**Table 1:** Summary of factors associated with long-term prognosis of patients with VSA by evidence degree.

Strong	Intermediate	Low	Not Related
* Serious clinical manifestations [5,9,35,45,46]	Age [18,19,20]	Obesity [22,30]	Sex [16,22,26]
Organic stenosis [5,13,17,22,24,37]	Smoking [34,35,36,38]	Alcohol [29,41]	Aspirin [74,75,76,77]
Definite spasm [17,52]	Long-acting nitrate (harmful) [55,64,65,66]	** Traditional risk factors [14,18,29]	
Multi-vessel spasm [28,29,35,53]		Myocardial bridging [61]	
Focal spasm [54,55,56,57]		Statin [45,57,69,70,71]	
Calcium channel blocker [5,13,18,35,63]		C-reactive protein [23,26,78]	
		Socioeconomic status [18]	

* Indicates ST elevation, acute coronary syndrome and sudden cardiac death, and ** indicates hypertension, diabetes mellitus and dyslipidemia. VSA, vasospastic angina; ACS, acute coronary syndrome.

## References

[1] Prinzmetal M., Kennamer R., Merliss R., Wada T., Bor N. (1959). Angina pectoris. I. A variant form of angina pectoris; preliminary report. Am. J. Med..

[2] Crea F., Lanza G.A. (2011). New light on a forgotten disease: Vasospastic angina. J. Am. Coll. Cardiol..

[3] Stern S., Bayes de Luna A. (2009). Coronary artery spasm: A 2009 update. Circulation.

[4] Yasue H., Kugiyama K. (1997). Coronary spasm: Clinical features and pathogenesis. Intern. Med..

[5] Yasue H., Takizawa A., Nagao M., Nishida S., Horie M., Kubota J., Omote S., Takaoka K., Okumura K. (1988). Long-term prognosis for patients with variant angina and influential factors. Circulation.

[6] Ludmer P.L., Selwyn A.P., Shook T.L., Wayne R.R., Mudge G.H., Alexander R.W., Ganz P. (1986). Paradoxical vasoconstriction induced by acetylcholine in atherosclerotic coronary arteries. N. Engl. J. Med..

[7] Yasue H., Nakagawa H., Itoh T., Harada E., Mizuno Y. (2008). Coronary artery spasm—Clinical features, diagnosis, pathogenesis, and treatment. J. Cardiol..

[8] Myerburg R.J., Kessler K.M., Mallon S.M., Cox M.M., de Marchena E., Interian A., Castellanos A. (1992). Life-threatening ventricular arrhythmias in patients with silent myocardial ischemia due to coronary-artery spasm. N. Engl. J. Med..

[9] Ahn J.M., Lee K.H., Yoo S.Y., Cho Y.R., Suh J., Shin E.S., Lee J.H., Shin D.I., Kim S.H., Baek S.H. (2016). Prognosis of variant angina manifesting as aborted sudden cardiac death. J. Am. Coll. Cardiol..

[10] Hung M.J., Cheng C.W., Yang N.I., Hung M.Y., Cherng W.J. (2007). Coronary vasospasm-induced acute coronary syndrome complicated by life-threatening cardiac arrhythmias in patients without hemodynamically significant coronary artery disease. Int. J. Cardiol..

[11] Beltrame J.F., Sasayama S., Maseri A. (1999). Racial heterogeneity in coronary artery vasomotor reactivity: Differences between Japanese and Caucasian patients. J. Am. Coll. Cardiol..

[12] Ong P., Athanasiadis A., Borgulya G., Mahrholdt H., Kaski J.C., Sechtem U. (2012). High prevalence of a pathological response to acetylcholine testing in patients with stable angina pectoris and unobstructed coronary arteries. The ACOVA Study (Abnormal coronary vasomotion in patients with stable angina and unobstructed coronary arteries). J. Am. Coll. Cardiol..

[13] Waters D.D., Miller D.D., Szlachcic J., Bouchard A., Méthé M., Kreeft J., Théroux P. (1983). Factors influencing the long-term prognosis of treated patients with variant angina. Circulation.

[14] Bory M., Pierron F., Panagides D., Bonnet J.L., Yvorra S., Desfossez L. (1996). Coronary artery spasm in patients with normal or near normal coronary arteries. Long-term follow-up of 277 patients. Eur. Heart J..

[15] Shimokawa H., Nagasawa K., Irie T., Egashira S., Egashira K., Sagara T., Kikuchi Y., Nakamura M. (1988). Clinical characteristics and long-term prognosis of patients with variant angina. A comparative study between western and Japanese populations. Int. J. Cardiol..

[16] Kawana A., Takahashi J., Takagi Y., Yasuda S., Sakata Y., Tsunoda R., Ogata Y., Seki A., Sumiyoshi T., Matsui M. (2013). Gender differences in the clinical characteristics and outcomes of patients with vasospastic angina—A report from the Japanese Coronary Spasm Association. Circ. J..

[17] Shin D.I., Baek S.H., Her S.H., Han S.H., Ahn Y., Park K.H., Kim D.S., Yang T.H., Choi D.J., Suh J.W. (2015). The 24-month prognosis of patients with positive or intermediate results in the intracoronary ergonovine provocation test. JACC Cardiovasc. Interv..

[18] Kim H.L., Kim J., Kim H.J., Lim W.H., Lee J.Y. (2017). Incidence and factors associated with mortality in 2476 patients with variant angina in Korea. Sci. Rep..

[19] Jones C.A., Perera A., Chow M., Ho I., Nguyen J., Davachi S. (2009). Cardiovascular disease risk among the poor and homeless—What we know so far. Curr. Cardiol. Rev..

[20] Kim H.L., Lee S.H., Kim J., Kim H.J., Lim W.H., Seo J.B., Chung W.Y., Kim S.H., Zo J.H., Kim M.A. (2016). Incidence and Risk factors associated with hospitalization for variant angina in Korea. Medicine (Baltim.).

[21] Choi W.G., Kim S.H., Rha S.W., Chen K.Y., Li Y.J., Choi B.G., Choi S.Y., Kim J.W., Kim E.J., Park C.G. (2016). Impact of old age on clinical and angiographic characteristics of coronary artery spasm as assessed by acetylcholine provocation test. J. Geriatr. Cardiol..

[22] Kim H.L., Jo S.H., Kim H.J., Lee M.H., Seo W.W., Baek S.H. (2020). Sex differences in clinical characteristics and long-term outcomes in patients with vasospastic angina: Results from the VA-Korea registry, a prospective multi-center cohort. Biol. Sex Differ..

[23] Hung M.J., Hsu K.H., Hu W.S., Chang N.C., Hung M.Y. (2013). C-reactive protein for predicting prognosis and its gender-specific associations with diabetes mellitus and hypertension in the development of coronary artery spasm. PLoS ONE.

[24] Kim H.J., Lee M.H., Jo S.H., Seo W.W., Kim H.L., Lee K.Y., Yang T.H., Her S.H., Han S.H., Lee B.K. (2021). Effect of significant coronary artery stenosis on prognosis in patients with vasospastic angina: A propensity score-matched analysis. J. Clin. Med..

[25] White H.D., Barbash G.I., Califf R.M., Simes R.J., Granger C.B., Weaver W.D., Kleiman N.S., Aylward P.E., Gore J.M., Vahanian A. (1996). Age and outcome with contemporary thrombolytic therapy. Results from the GUSTO-I trial. Global Utilization of streptokinase and TPA for occluded coronary arteries trial. Circulation.

[26] Lee D.H., Park T.K., Seong C.S., Gwag H.B., Lim A.Y., Oh M.S., Cho S.W., Yang J.H., Song Y.B., Hahn J.Y. (2017). Gender differences in long-term clinical outcomes and prognostic factors in patients with vasospastic angina. Int. J. Cardiol..

[27] Walling A., Waters D.D., Miller D.D., Roy D., Pelletier G.B., Théroux P. (1987). Long-term prognosis of patients with variant angina. Circulation.

[28] Takagi Y., Takahashi J., Yasuda S., Miyata S., Tsunoda R., Ogata Y., Seki A., Sumiyoshi T., Matsui M., Goto T. (2013). Prognostic stratification of patients with vasospastic angina: A comprehensive clinical risk score developed by the Japanese Coronary Spasm Association. J. Am. Coll. Cardiol..

[29] Han S.H., Lee K.Y., Her S.H., Ahn Y., Park K.H., Kim D.S., Yang T.H., Choi D.J., Suh J.W., Kwon H.M. (2019). Impact of multi-vessel vasospastic angina on cardiovascular outcome. Atherosclerosis.

[30] Lee M.H., Jo S.H., Kwon S., Park B.W., Bang D.W., Hyon M.S., Baek S.H., Han S.H., Her S.H., Shin D.I. (2020). Impact of overweight/obesity on clinical outcomes of patient with vasospastic angina: From the vasospastic angina in korea registry. Sci. Rep..

[31] Winniford M.D., Wheelan K.R., Kremers M.S., Ugolini V., van den Berg E., Niggemann E.H., Jansen D.E., Hillis L.D. (1986). Smoking-induced coronary vasoconstriction in patients with atherosclerotic coronary artery disease: Evidence for adrenergically mediated alterations in coronary artery tone. Circulation.

[32] Sugiishi M., Takatsu F. (1993). Cigarette smoking is a major risk factor for coronary spasm. Circulation.

[33] Powell J.T. (1998). Vascular damage from smoking: Disease mechanisms at the arterial wall. Vasc. Med..

[34] Choi B.G., Rha S.W., Park T., Choi S.Y., Byun J.K., Shim M.S., Xu S., Li H., Park S.H., Park J.Y. (2016). Impact of cigarette smoking: A 3-year clinical outcome of vasospastic angina patients. Korean Circ. J..

[35] Takagi Y., Yasuda S., Tsunoda R., Ogata Y., Seki A., Sumiyoshi T., Matsui M., Goto T., Tanabe Y., Sueda S. (2011). Clinical characteristics and long-term prognosis of vasospastic angina patients who survived out-of-hospital cardiac arrest: Multicenter registry study of the Japanese Coronary Spasm Association. Circ. Arrhythm. Electrophysiol..

[36] Figueras J., Domingo E., Ferreira I., Lidón R.M., Garcia-Dorado D. (2012). Persistent angina pectoris, cardiac mortality and myocardial infarction during a 12 year follow-up in 273 variant angina patients without significant fixed coronary stenosis. Am. J. Cardiol..

[37] Hao K., Takahashi J., Kikuchi Y., Suda A., Sato K., Sugisawa J., Tsuchiya S., Shindo T., Nishimiya K., Ikeda S. (2021). Prognostic impacts of comorbid significant coronary stenosis and coronary artery spasm in patients with stable coronary artery disease. J. Am. Heart Assoc..

[38] Szeto C.C., Kwan B.C., Chow K.M., Leung C.B., Law M.C., Li P.K. (2012). Prognostic value of arterial pulse wave velocity in peritoneal dialysis patients. Am. J. Nephrol..

[39] Oda H., Suzuki M., Oniki T., Kishi Y., Numano F. (1994). Alcohol and coronary spasm. Angiology.

[40] Takizawa A., Yasue H., Omote S., Nagao M., Hyon H., Nishida S., Horie M. (1984). Variant angina induced by alcohol ingestion. Am. Heart J..

[41] Sohn S.M., Choi B.G., Choi S.Y., Byun J.K., Mashaly A., Park Y., Jang W.Y., Kim W., Choi J.Y., Park E.J. (2018). Impact of alcohol drinking on acetylcholine-induced coronary artery spasm in Korean populations. Atherosclerosis.

[42] Mahmood S.S., Levy D., Vasan R.S., Wang T.J. (2014). The framingham heart study and the epidemiology of cardiovascular disease: A historical perspective. Lancet.

[43] Greenland P., Knoll M.D., Stamler J., Neaton J.D., Dyer A.R., Garside D.B., Wilson P.W. (2003). Major risk factors as antecedents of fatal and nonfatal coronary heart disease events. JAMA.

[44] Takatsu F., Watarai M. (2011). Mild stenosis makes prognosis of vasospastic angina worse. Coron. Artery Dis..

[45] Ishii M., Kaikita K., Sato K., Yamanaga K., Miyazaki T., Akasaka T., Tabata N., Arima Y., Sueta D., Sakamoto K. (2016). Impact of statin therapy on clinical outcome in patients with coronary spasm. J. Am. Heart Assoc..

[46] Cho S.W., Park T.K., Gwag H.B., Lim A.Y., Oh M.S., Lee D.H., Seong C.S., Yang J.H., Song Y.B., Hahn J.Y. (2016). Clinical outcomes of vasospastic angina patients presenting with acute coronary syndrome. J. Am. Heart Assoc..

[47] Yamashina Y., Yagi T., Namekawa A., Ishida A., Mibiki Y., Sato H., Nakagawa T., Sakuramoto M., Sato E., Komatsu J. (2014). Favorable outcomes of patients with vasospastic angina associated with cardiac arrest. J. Cardiol..

[48] Matsue Y., Suzuki M., Nishizaki M., Hojo R., Hashimoto Y., Sakurada H. (2012). Clinical implications of an implantable cardioverter-defibrillator in patients with vasospastic angina and lethal ventricular arrhythmia. J. Am. Coll. Cardiol..

[49] Meune C., Joly L.M., Chiche J.D., Charpentier J., Leenhardt A., Rozenberg A., Carli P., Sauval P., Weber S., Cracan A. (2003). Diagnosis and management of out-of-hospital cardiac arrest secondary to coronary artery spasm. Resuscitation.

[50] Chevalier P., Dacosta A., Defaye P., Chalvidan T., Bonnefoy E., Kirkorian G., Isaaz K., Denis B., Touboul P. (1998). Arrhythmic cardiac arrest due to isolated coronary artery spasm: Long-term outcome of seven resuscitated patients. J. Am. Coll. Cardiol..

[51] Miwa K., Kambara H., Kawai C. (1981). Exercise-induced angina provoked by aspirin administration in patients with variant angina. Am. J. Cardiol..

[52] Sunagawa O., Shinzato Y., Touma T., Tomori M., Fukiyama K. (2000). Differences between coronary hyperresponsiveness to ergonovine and vasospastic angina. Jpn. Heart J..

[53] Onaka H., Hirota Y., Shimada S., Kita Y., Sakai Y., Kawakami Y., Suzuki S., Kawamura K. (1996). Clinical observation of spontaneous anginal attacks and multivessel spasm in variant angina pectoris with normal coronary arteries: Evaluation by 24-hour 12-lead electrocardiography with computer analysis. J. Am. Coll. Cardiol..

[54] Sato K., Kaikita K., Nakayama N., Horio E., Yoshimura H., Ono T., Ohba K., Tsujita K., Kojima S., Tayama S. (2013). Coronary vasomotor response to intracoronary acetylcholine injection, clinical features, and long-term prognosis in 873 consecutive patients with coronary spasm: Analysis of a single-center study over 20 years. J. Am. Heart Assoc..

[55] Kim D.W., Her S.H., Ahn Y., Shin D.I., Han S.H., Kim D.S., Choi D.J., Kwon H.M., Gwon H.C., Jo S.H. (2018). Clinical outcome according to spasm type of single coronary artery provoked by intracoronary ergonovine tests in patients without significant organic stenosis. Int. J. Cardiol..

[56] Park Y.M., Han S.H., Ko K.P., Koh K.K., Kang W.C., Lee K., Shin K.C., Suh S.Y., Ahn T.H., Choi I.S. (2013). Diffuse multi-vessel coronary artery spasm: Incidence and clinical prognosis. Int. J. Cardiol..

[57] Seo W.W., Jo S.H., Kim S.E., Han S.H., Lee K.Y., Her S.H., Lee M.H., Cho S.S., Baek S.H. (2020). Clinical impact of statin therapy on vasospastic angina: Data from a Korea nation-wide cohort study. Heart Vessel..

[58] Ishii M., Kaikita K., Sato K., Tanaka T., Sugamura K., Sakamoto K., Izumiya Y., Yamamoto E., Tsujita K., Yamamuro M. (2015). Acetylcholine-provoked coronary spasm at site of significant organic stenosis predicts poor prognosis in patients with coronary vasospastic angina. J. Am. Coll. Cardiol..

[59] Teragawa H., Fukuda Y., Matsuda K., Hirao H., Higashi Y., Yamagata T., Oshima T., Matsuura H., Chayama K. (2003). Myocardial bridging increases the risk of coronary spasm. Clin. Cardiol..

[60] Kim J.W., Seo H.S., Na J.O., Suh S.Y., Choi C.U., Kim E.J., Rha S.W., Park C.G., Oh D.J. (2008). Myocardial bridging is related to endothelial dysfunction but not to plaque as assessed by intracoronary ultrasound. Heart.

[61] Nam P., Choi B.G., Choi S.Y., Byun J.K., Mashaly A., Park Y., Jang W.Y., Kim W., Choi J.Y., Park E.J. (2018). The impact of myocardial bridge on coronary artery spasm and long-term clinical outcomes in patients without significant atherosclerotic stenosis. Atherosclerosis.

[62] Kim S.E., Jo S.H., Han S.H., Lee K.Y., Her S.H., Lee M.H., Seo W.W., Cho S.S., Baek S.H. (2021). Comparison of calcium-channel blockers for long-term clinical outcomes in patients with vasospastic angina. Korean J. Intern. Med..

[63] Lette J., Gagnon R.M., Lemire J.G., Morissette M. (1984). Rebound of vasospastic angina after cessation of long-term treatment with nifedipine. Can. Med. Assoc. J..

[64] Takahashi J., Nihei T., Takagi Y., Miyata S., Odaka Y., Tsunoda R., Seki A., Sumiyoshi T., Matsui M., Goto T. (2015). Prognostic impact of chronic nitrate therapy in patients with vasospastic angina: Multicentre registry study of the Japanese coronary spasm association. Eur. Heart J..

[65] Kosugi M., Nakagomi A., Shibui T., Kato K., Kusama Y., Atarashi H., Mizuno K. (2011). Effect of long-term nitrate treatment on cardiac events in patients with vasospastic angina. Circ. J..

[66] Kim C.H., Park T.K., Cho S.W., Oh M.S., Lee D.H., Seong C.S., Gwag H.B., Lim A.Y., Yang J.H., Song Y.B. (2018). Impact of different nitrate therapies on long-term clinical outcomes of patients with vasospastic angina: A propensity score-matched analysis. Int. J. Cardiol..

[67] Ray K.K., Cannon C.P. (2005). The potential relevance of the multiple lipid-independent (pleiotropic) effects of statins in the management of acute coronary syndromes. J. Am. Coll. Cardiol..

[68] Oesterle A., Laufs U., Liao J.K. (2017). Pleiotropic effects of statins on the cardiovascular system. Circ. Res..

[69] Yasue H., Mizuno Y., Harada E., Itoh T., Nakagawa H., Nakayama M., Ogawa H., Tayama S., Honda T., Hokimoto S. (2008). Effects of a 3-hydroxy-3-methylglutaryl coenzyme A reductase inhibitor, fluvastatin, on coronary spasm after withdrawal of calcium-channel blockers. J. Am. Coll. Cardiol..

[70] Oh M.S., Yang J.H., Lee D.H., Park T.K., Song Y.B., Hahn J.Y., Choi J.H., Lee S.H., Gwon H.C., Choi S.H. (2016). Impact of statin therapy on long-term clinical outcomes of vasospastic angina without significant stenosis: A propensity-score matched analysis. Int. J. Cardiol..

[71] Park S.J., Park H., Kang D., Park T.K., Park J., Cho J., Chung C.R., Jeon K., Guallar E., Cho J. (2019). Association of statin therapy with clinical outcomes in patients with vasospastic angina: Data from Korean health insurance review and assessment service. PLoS ONE.

[72] Miwa K., Kambara H., Kawai C. (1979). Variant angina aggravated by aspirin. Lancet.

[73] Park J.Y., Rha S.W., Poddar K.L., Ramasamy S., Chen K.Y., Li Y.J., Choi B.G., Ryu S.K., Choi J.W., Park S.H. (2012). Impact of low-dose aspirin on coronary artery spasm as assessed by intracoronary acetylcholine provocation test in Korean patients. J. Cardiol..

[74] Lim A.Y., Park T.K., Cho S.W., Oh M.S., Lee H.D., Seong C.S., Gwag H.B., Yang J.H., Song Y.B., Hahn J.Y. (2016). Clinical implications of low-dose aspirin on vasospastic angina patients without significant coronary artery stenosis; a propensity score-matched analysis. Int. J. Cardiol..

[75] Cho S.S., Jo S.H., Han S.H., Lee K.Y., Her S.H., Lee M.H., Seo W.W., Kim S.E., Yang T.H., Park K.H. (2019). Clopidogrel plus aspirin use is associated with worse long-term outcomes, but aspirin use alone is safe in patients with vasospastic angina: Results from the VA-Korea registry, a prospective multi-center cohort. Sci. Rep..

[76] Ishii M., Kaikita K., Sato K., Yamanaga K., Miyazaki T., Akasaka T., Tabata N., Arima Y., Sueta D., Sakamoto K. (2016). Impact of aspirin on the prognosis in patients with coronary spasm without significant atherosclerotic stenosis. Int. J. Cardiol..

[77] Lin Y., Chen Y., Yuan J., Qin H., Dong S., Chen Q. (2021). Impact of aspirin use on clinical outcomes in patients with vasospastic angina: A systematic review and meta-analysis. BMJ Open.

[78] Park J.Y., Rha S.W., Li Y.J., Chen K.Y., Choi B.G., Choi S.Y., Ryu S.K., Choi J.W., Kim T.K., Kim J.M. (2013). The impact of high sensitivity C-reactive protein level on coronary artery spasm as assessed by intracoronary acetylcholine provocation test. Yonsei Med. J..

[79] Hung M.J., Cherng W.J., Yang N.I., Cheng C.W., Li L.F. (2005). Relation of high-sensitivity C-reactive protein level with coronary vasospastic angina pectoris in patients without hemodynamically significant coronary artery disease. Am. J. Cardiol..

[80] Hung M.J., Cherng W.J. (2013). Coronary vasospastic angina: Current understanding and the role of inflammation. Acta Cardiol. Sin..

[81] Sung J., Song Y.M., Hong K.P. (2020). Relationship between the shift of socioeconomic status and cardiovascular mortality. Eur. J. Prev. Cardiol..

[82] González M.A., Artalejo R.F., Calero J.R. (1998). Relationship between socioeconomic status and ischaemic heart disease in cohort and case-control studies: 1960–1993. Int. J. Epidemiol..

[83] Kaplan G.A., Keil J.E. (1993). Socioeconomic factors and cardiovascular disease: A review of the literature. Circulation.

